# Exciton Bimolecular Annihilation Dynamics in Push–Pull Semiconductor Polymers

**DOI:** 10.1021/acs.jpclett.3c03094

**Published:** 2024-01-02

**Authors:** Yulong Zheng, Rahul Venkatesh, Esteban Rojas-Gatjens, Elsa Reichmanis, Carlos Silva-Acuña

**Affiliations:** † School of Chemistry and Biochemistry, Georgia Institute of Technology, 901 Atlantic Drive, Atlanta, Georgia 30332, United States; ‡ School of Chemical and Biomolecular Engineering, Georgia Institute of Technology, 311 Ferst Drive NW, Atlanta, Georgia 30332, United States; ¶ Department of Chemical & Biomolecular Engineering, Lehigh University, 124 E. Morton Street, Bethlehem, Pennsylvania 18015, United States; § Institut Courtois & Département de physique, Université de Montréal, 1375 Avenue Thérèse-Lavoie-Roux, Montréal H2V 0B3, Québec, Canada

## Abstract

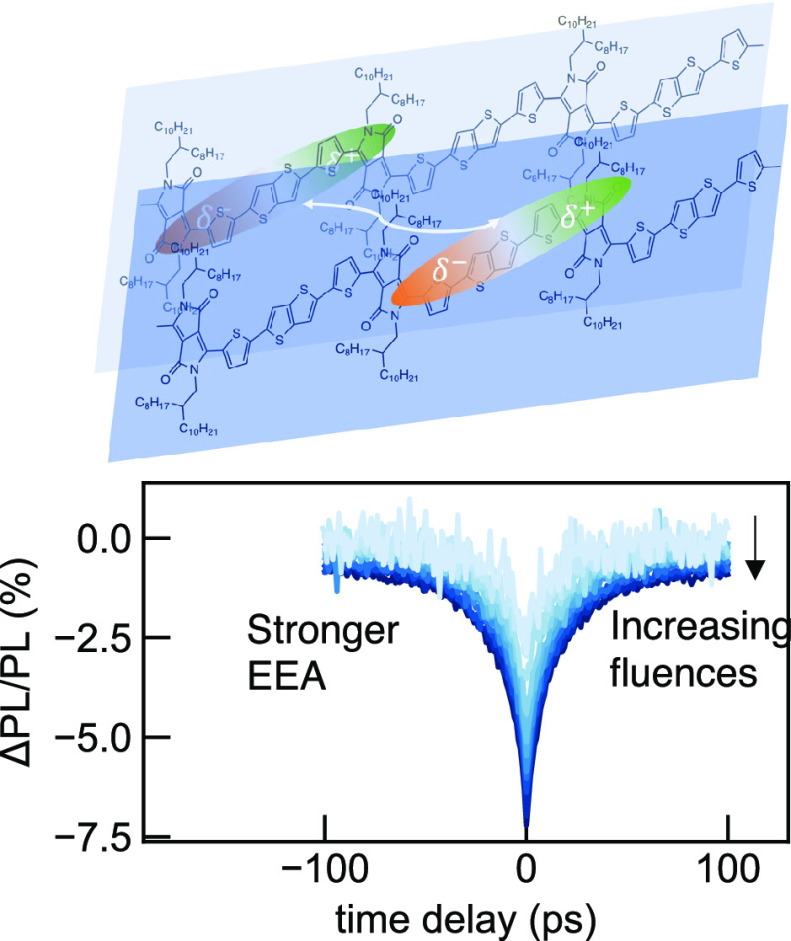

Exciton–exciton annihilation is a ubiquitous nonlinear dynamic phenomenon in materials hosting Frenkel excitons. In this work, we investigate the nonlinear exciton dynamics of an electron push–pull conjugated polymer by fluence-dependent transient absorption and excitation-correlation photoluminescence spectroscopy, where we can quantitatively show the latter to be a more selective probe of the nonlinear dynamics. Simulations based on a time-independent exciton annihilation model show a decreasing trend for the extracted annihilation rates with excitation fluence. Further investigation of the fluence-dependent transients suggests that the exciton–exciton annihilation bimolecular rates are not constant in time, displaying a *t*^–1/2^ time dependence, which we rationalize as reflective of one-dimensional exciton diffusion, with a diffusion length estimated to be 9 ± 2 nm. In addition, exciton annihilation gives rise to a long-lived species that recombines on a nanosecond time scale. Our conclusions shed broad light onto nonlinear exciton dynamics in push–pull conjugated polymers.

Frenkel excitons are the primary photoexcitations in conjugated polymers. Following the vertical transitions, excitons experience ultrafast electronic and conformational relaxation to the local minima of the exciton band.^1−5^ During this process, a very small percent of the population may dissociate to form polaron pairs in neat conjugated polymer thin films, even in the absence of successive two-quantum excitation.^6^ Thereafter, excitons can be transported through incoherent energy hopping.^7,8^ When the samples are exposed to sufficiently high laser fluence, high exciton densities may give rise to singlet exciton–exciton annihilation (EEA). In this work, we probe the EEA dynamics in a conjugated push–pull polymer by comparing transient absorption (TA) and excitation correlation photoluminescence (ECPL) spectroscopic measurements. With a time-independent annihilation model, both trends of the annihilation rates appear to decrease with fluence before a plateau is reached. Previously, the Franck–Condon analysis performed on the absorption line shape of the same samples prepared from a variety of precursor-solution concentrations revealed an increasing trend of chain backbone order with the viscosity of the precursor solution.^9^ In this Letter, we report that thin films prepared from higher precursor-solution concentrations show higher annihilation rates, likely due to short-range Coulombic interactions and/or wave function overlap enhanced by the chain planarization identified previously. Further investigation of the time evolution of exciton density at an early time (20 ps) in TA indicates that the annihilation rate has a *t*^–1/2^ dependence, suggesting that exciton diffusion in the push–pull conjugated polymer DPP-DTT (poly[2,5-(2-octyldodecyl)-3,6-diketopyrrolopyrrole-*alt*-5,5-(2,5-di(thien-2-yl)thieno-[3,2-*b*]-thiophene)]) is one-dimensional. In addition to the short-time decay trace, the long-lived tail prevails with increasing pumping fluences, which shows a quadratic dependence, indicating an increasing yield of charges through EEA.

Previously, two mechanisms have been proposed to explain the annihilation process. One is that the annihilation is achieved through Förster-type long-range Coulombic interaction.^10^ Due to the random spatial distribution of excitons, the ensemble-averaged annihilation rates will decrease with time.^8,11,12^ Another model considers the anisotropy of exciton diffusion^13,14^ and excitons can only interact when they are in proximity, either through short-range Coulombic interaction or wavefunction overlap.^7,15^ In either scenario, the temporal dependence of the annihilation rates reflects the spatial dependence of the exciton distribution or their motion. Despite the fact that the pump fluences used in these measurements are orders of magnitude higher than the solar power, the extracted annihilation parameter with the fluence dependence could be theoretically extrapolated to a fluence-independent value, which suggests the ability of intrinsic exciton diffusion. Subsequent to annihilation, one exciton gets deexcited to the ground state, while the other is promoted to a higher excited state. While energy relaxation to the low-lying excited state could still occur, the probability of the high-lying excited state dissociating to polaron pairs also increases.^16^ Therefore, new long-lived excited species could also be observed with increasing pump fluences.^17^

The nonlinearity and temporal dependence of EEA processes distort the monoexponential dynamics on a picosecond time scale in common time-resolved measurements, such as transient absorption (TA) and time-resolved photoluminescence (PL).^13,15,18−21^ The mixing of the natural monoexponential decay, EEA, and other linear photophysical processes prohibits the isolation of nonlinear processes from the temporally resolved signals. In comparison, excitation-correlation (EC) spectroscopy can provide a more selective response to nonlinear dynamics such as EEA due to phase-sensitive detection of two-pulse excitation. EC spectroscopy employs two laser beam replicas, each modulated with one chopper at a slightly different frequency.^22,23^ Therefore, the linear PL from each channel can be acquired when demodulating at each reference frequency. Furthermore, nonlinear population mixing arising from EEA induced by the two pulses can be acquired when the signal is demodulated at the sum of frequencies. Commonly, the EC signals, ΔPL/PL, are demonstrated as a proportion of the nonlinear signal from the sum of nonlinear and linear signals from all three demodulation channels. With the relative arrival time between the two beams controlled by a delay stage, the evolution of the nonlinear dynamics can be mapped. Although excitation correlation photoluminescence (ECPL) and photocurrent (PC) techniques are not as widely used as TA or time-resolved PL, their application has provided new insight on the photophysics of inorganic semiconductors,^24−26^ carbon nanotubes,^27,28^ two-dimensional transition-metal dichalcogenides^29^ and hybrid organic–inorganic perovskites^30−33^ due to their sensitivity to nonlinear photophysical responses. Of particular relevance to organic semiconductors, Rojas-Gatjens et al. recently investigated the nonlinear PL and PC responses of an organic small-molecule photovoltaic material, where the dominant source of charge-carrier generation is ascribed to the EEA process.^34^ Compared to the conjugated homopolymers, conjugated push–pull copolymers inherit strong charge-transfer character due to the differences in the electronegativities of the electron-deficient and -sufficient domains, which could contribute to the driving force for EEA.^35^ Here, our work provides new insights into exciton diffusion in conjugated push–pull polymers by comparing the TA and ECPL measurements, experimentally and via modeling, which can be further developed in new optoelectronic systems.

We focus on a push–pull conjugated polymer, DPP-DTT (Figure 1a), following previous ultrafast measurements on this material.^9^ A series of samples prepared from precursor solutions of 4, 6, and 8 g/L in chlorobenzene were cast by using the blade-coating technique. The detailed sample preparation process and characterization are described elsewhere.^36^ The absorption spectra in Figure 1b show that the vibronic ratio of 0–0 and 0–1 transition decreases with increasing concentration, suggesting increasing interchain excitonic interactions.^9^ To probe the exciton dynamics, fluence-dependent TA measurements are first performed under an excitation wavelength of 730 nm. Here, measurements of the 8 g/L sample under the lowest and highest fluence are displayed in Figure 1c,d, respectively. The other TA measurements with intermediate fluences are also shown in Figure S1 in the Supporting Information. Both measurements show similar spectral responses with strong ground-state bleaching (GSB) from 1.4 to 1.9 eV and photoinduced absorption (PIA) beyond 1.4 eV. It is worth pointing out that the probe temporal dependence of the TA signal at higher pumping fluence shows a weak, long-lived species, which will be examined in more detail later. The temporal cuts of the spectra are also shown correspondingly in Figure 1e,f. A small spectral shift (less than 10 meV) is noticed between the two fluences, which could be ascribed to the induced electric field under excessive exciton densities.^37^ The decay traces are further examined at 750 nm within the GSB region, where the oscillator strengths stem from the 0–0 vibronic origin. We assume that the primary PL and GSB share the same dynamics since only the first excited states are mostly populated. Such an assumption allows the following EEA equations to be applicable to both TA and ECPL spectroscopies.

**Figure 1:**
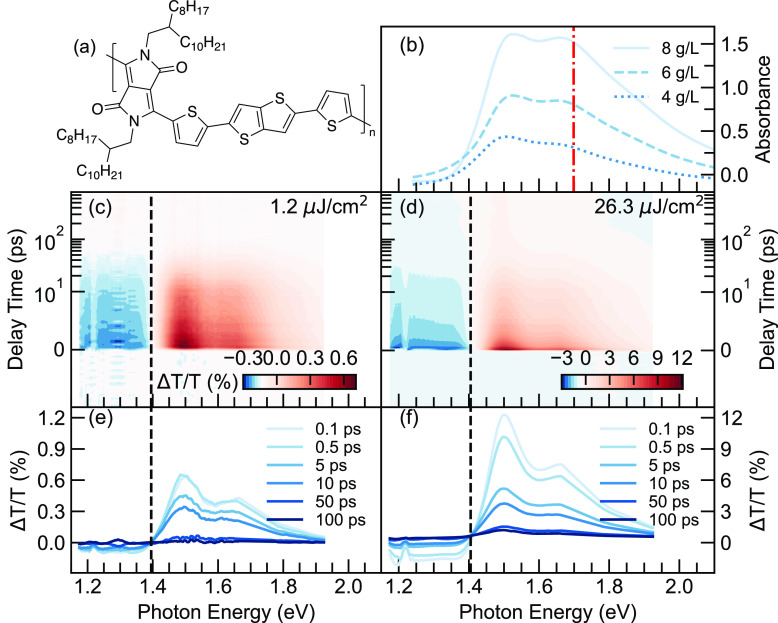
(a) Molecular structure of the repeating unit of DPP-DTT. (b) The absorption spectra of the DPP-DTT thin films prepared from precursor solutions of 4 (dotted), 6 (dashed), and 8 (solid) g/L. The red dot–dashed line indicates the pump wavelength used in TA and ECPL measurements.(c and d) Transient absorption maps for samples of 8 g/L, excited by the pump wavelength of (730 nm or 1.70 eV) under low and high fluence, respectively. The 1.2 and 26.3 μJ/cm^2^ corresponds to the excitation density of 3.8 × 10^17^ and 8.3 × 10^18^ cm^–3^ (see the Supporting Information for experimental details). (e and f) Temporal cuts for the spectra. The dashed line is a guide for the eye to determine the zero cross point.

To account for the exciton decay trace, a simple bimolecular exciton–exciton annihilation decay equation reads as
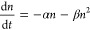
1where α is the monomolecular exciton decay constant, while β denotes the EEA rate constant. It is worth noting that eq 1 assumes that the natural exciton decay and time-independent EEA process are the only two primary pathways for exciton decay that contribute to the final PL signals, whereas secondary dynamic processes and excited-state species could also contribute in reality.^21,38^ For example, charge-transfer excitons could be generated either directly^37,39−41^ or through exciton dissociation from a higher-energy excited state.^6^ Charge recombination could give rise to delayed PL with power-law time dependence.^21,38^ Nonetheless, the primary excitation dominates the majority of the PL signals, and the EEA mechanism should serve as the simplest quantitative case study. The equation has an analytical expression
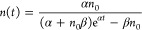
2Equation 2 can be further linearized as^19,42^
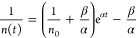
3The initial excitation density is given as *n*_0_ upon excitation. A quick examination of eq 3 shows that the inverse of the excitation density should have a negative intercept.

To extract the bimolecular annihilation rate, β in the form of eq 3, the fluence-dependent temporal cuts at 750 nm from TA are plotted in Figure 2a. At relatively low fluences, the log-scale differential transmission traces show a mostly linear dependence on delay time, while within 20 ps, the nonlinear decaying component due to EEA becomes more prevalent. The monoexponential decay rate α is fixed at 0.053 ps^–1^ as exctrated from an exponential fit, excited by the lowest pump fluence (1.2 μJ/cm^2^), which is assumed to be in the regime of dominant monoexponential decay. Therefore, β can be acquired by solving the slope and intercept of the linear fit together, as shown in Figure 2b. Before we move on to discussing the acquired annihilation rates, it is worth pointing out that the extraction of the annihilation rates relies on the assumption that the initial differential signal is attributed to a single-step pumping excitation. As shown by Silva et al.,^6^ two-step excitation originating from the leading and trailing edge of a single pulse could also lead to nonlinear decaying dynamics in TA, which mixes with the EEA source. However, as shown in Figure 2c, the differential transmission signals at time zero not only have a linear dependence on the excitation density but also have an almost 0 *y*-intercept (0.042 ± 0.183), which excludes the possibility of two-step excitation. Based on eq 3, the annihilation rates can be readily calculated since α is known and *n*_0_ can be estimated with laser fluence, film thickness, and absorption coefficients.

**Figure 2:**
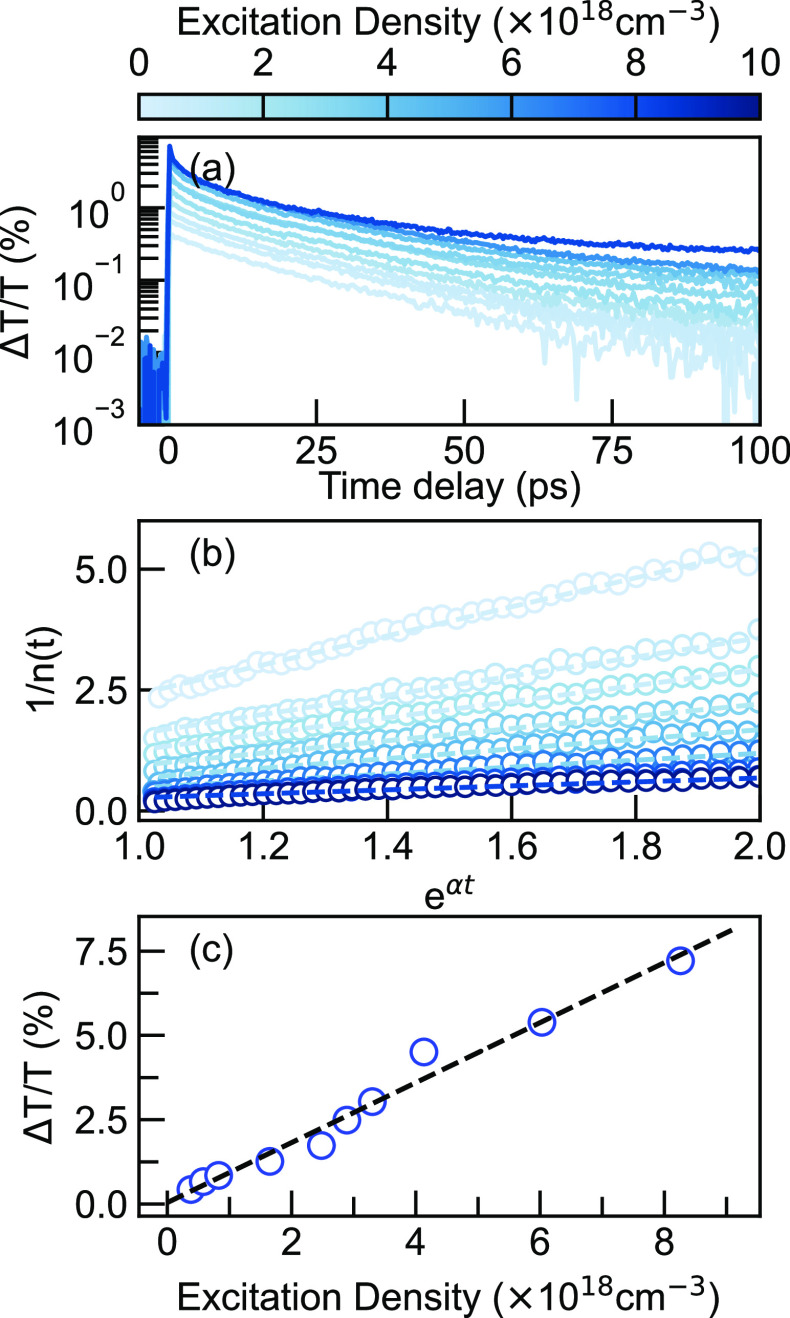
(a) TA Decays at 750 nm with varying excitation densities. With increasing excitation densities, a faster decay is observed within the first few picoseconds. (b) Linearized TA decays (white open circles) fit to eq 3 (dashed straight lines). The first 20 ps is chosen and converted for the exponential *x*-axis. (c) The dependence of initial differential transmission (open circles) on excitation densities. The black dashed line fits the linear relationship with a slope of 0.889(±0.047) and intercept of 0.042(±0.183).

The annihilation rates acquired from TA measurements can be further compared to those of their ECPL counterparts. Prior to that, we resort to deriving an annihilation-based model in describing the ECPL signal profiles. Previous work revealed that with samples prepared from higher-concentration solutions, polymer interchain excitonic interaction increases, as well as the chain backbone planarity.^9,36^ Both factors might contribute to a distinct strength of the exciton–exciton interaction. With the aforementioned ECPL working principle, all ECPL profiles measured on DPP-DTT thin films of different precursor concentrations demonstrate a negative signal and diminish with delayed times between the two pulses, as shown in Figure 3. A detailed description of the ECPL setup can be found in the Supporting Information. The overall negative signals reflect EEA as an efficient linear PL quenching pathway, while the decaying nonlinear signals originate from the less temporal overlap between the two pulses, thus less sufficient population mixing. To analyze the results quantitatively, we further implement eq 2 based on lock-in detection, which essentially gives rise to a time-integrated signal
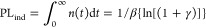
4where γ is a unitless parameter defined as . Considering that the monoexponential decay is constant, the product of the initial excitation density and annihilation rate, thus γ, is a measure of the strength of the EEA process. On the other hand, the nonlinear signal demodulated at the sum of the chopping frequencies depends on the delay time between the two beams. The contribution of nonlinear dynamics to the integrated PL intensity depends on the delay between the two excitation pulses since the exciton density generated by each pulse depends on time. The total amount of excitons should be given as the sum of the residual from the first decay and the newly generated amount
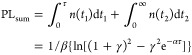
5Eventually, the experimentally meaningful equation can be given as
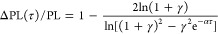
6One extreme scenario can be readily inspected: when the time delay τ approaches infinity, eq 6 will give 0, indicating null PL signal arising from nonlinear dynamics, which is expected as the long intervals between the two pulses prohibit the generation of the cross term. As indicated earlier, ECPL is more selective in separating nonlinear signals than TA. This can be readily seen if we assume no annihilation, suggesting that the excitation should be completely monoexponential. It then can be shown that PL_sum_ is simply double PL_ind_, which is . Therefore, eq 6 will yield 0, which rigorously shows that linear dynamics alone would not give ECPL signals.

**Figure 3:**
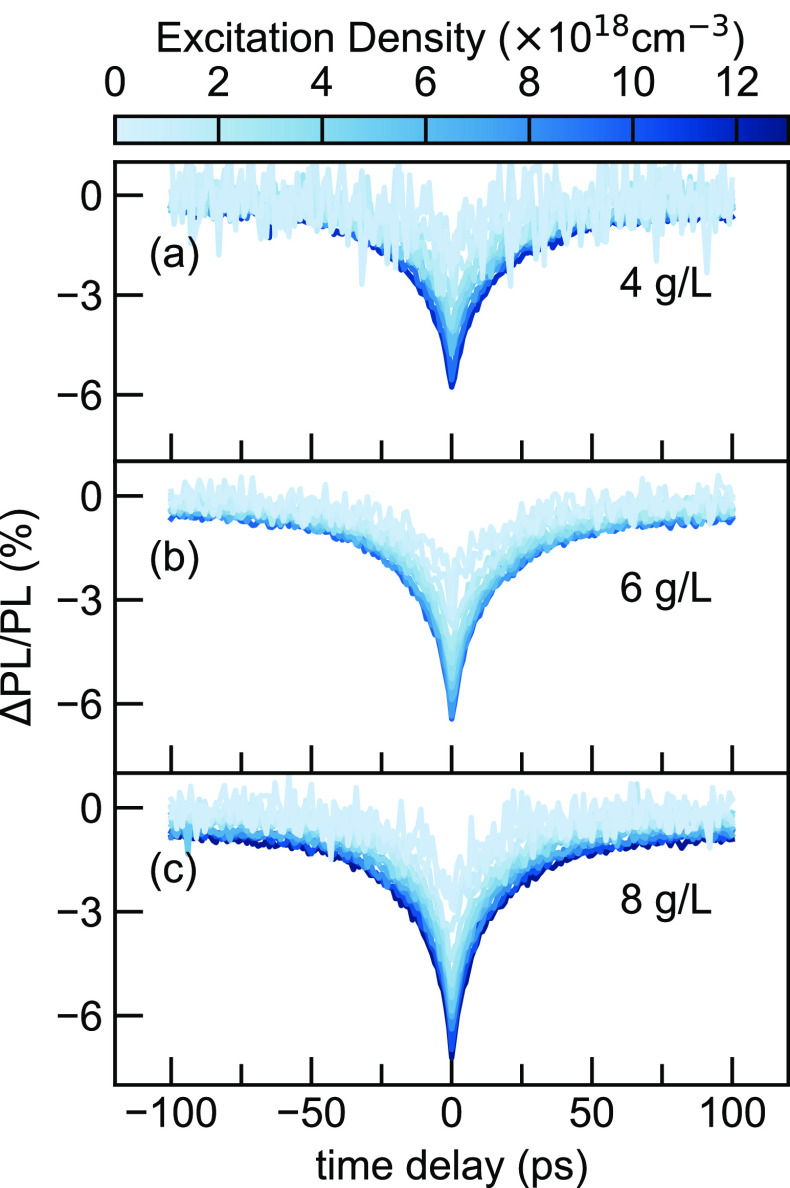
ECPL nonlinear response profiles excited at 730 nm pump for samples prepared from 4 (a), 6 (b), and 8 g/L (c) precursor solutions. The PL signals were filtered to collect the wavelength range of 750–1100 nm. The measurements are performed under a variation of excitation densities coded by the colorbar scale.

The complete simulation results are shown in Figures S3–S5, which demonstrate excellent consistency with the experimental results. The extracted γ with increasing excitation densities implies stronger EEA effects as expected (Figure 4a). Interestingly, the γ values acquired from the sample of 4 g/L are notably lower than those prepared from higher precursor concentrations. Furthermore, simulations based on eq 6 yield annihilation rates on the order of magnitude of 1 × 10^–9^ cm^–3^ s^–1^ (Figure 4b). Meanwhile, the annihilation rates extracted from TA also show a decreasing trend with excitation density even with overall higher β values, as shown in Figure 4c. Indeed, annihilation rates acquired from time-integrated measurements are frequently shown to be lower compared to the parameters extracted from their time-resolved counterparts for the same type of conjugated polymer.^43,44^ Such difference might be partially ascribed to integrating long-lived PL signals that originate from polaron-pair recombination and/or triplet–triplet annihilation.^21^ Those long-lived PL signals compensate for the PL quenching by EEA in that annihilation rates are underestimated with higher pumping fluences. Except for slight differences in the absolute values of β, the annihilation rates show a consistent asymptotic decreasing trend. It is worth mentioning that decreasing annihilation rates are not uncommonly observed. Previous literature ascribed the origins to either excitons generated within the EEA radius annihilating rapidly or excitons with a shorter effective lifetime under higher densities.^19,44^ Nevertheless, excitons generated within the annihilation radius should not be rare even under low excitation fluences, as the interaction radius is calculated as an ensemble average. On the other hand, the effective monomolecular lifetime would shorten due to stimulated emission or excited-state absorption with increasing fluence; their variations are much smaller in contrast to the change of γ (see Figure S6). Alternatively, it is worth pointing out that the annihilation rate could be a time-dependent value, especially in the early stage.^11^ Previous publications indicate that such dependence originates from the dimensionality of exciton diffusion, where not only isotropic but also one- and two-dimensional diffusion have been identified in different semiconductor polymers, which might be accountable for the decreasing trend for the annihilation rates with fluences.^13,14,42^

**Figure 4:**
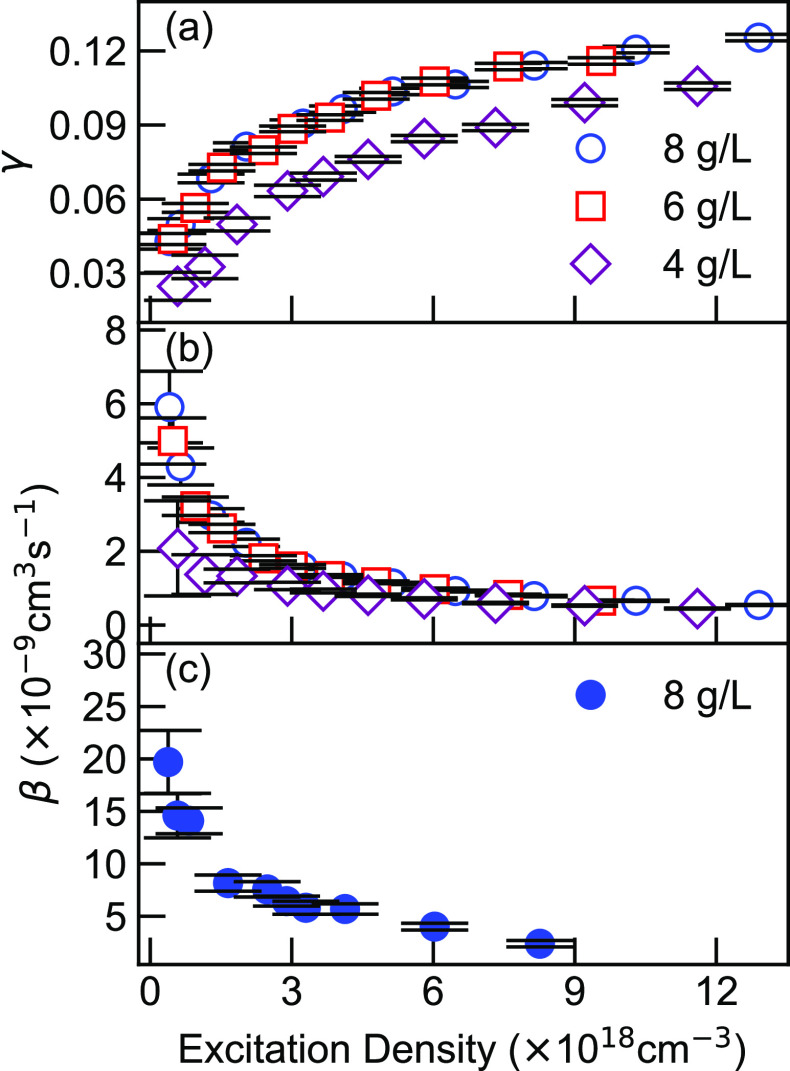
Excitation-density dependence in DPP-DTT thin films prepared from 4, 6, and 8 g/L solutions of (a) unitless parameter, γ, and (b) EEA rates, β, acquired by fitting ECPL profiles using eq 4 compared with (c) EEA rates acquired from Figure 3b measured from TA.

The exciton annihilation rate could have a *t*^–1/2^ time dependence due to either the spatial distribution of excitons, which annihilate through long-range Coulombic interactions, or one-dimensional diffusion-limited annihilation. In either scenario, the time-dependent annihilation model (eq 2) could be reformulated as^45^
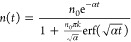
7where *k* ≡ β(*t*) × √*t* so that the newly defined annihilation rate constant, *k*, can now be simply described as a time-independent term and erf is the error function. For a better comparison, all simulations based on monoexponential, time-independent, and time-dependent models are shown in the lowest and highest TA decay traces in Figure 5a,b, respectively. Under the lowest pumping fluence, all three models fit the dynamics closely, indicating that the dynamics at low pump fluence is dominated by monoexponential decay with minor impact from EEA. However, under high pump fluence, a small deviation becomes clear in the early delay times (first 2 ps) when comparing the time-dependent annihilation model with the other two; the first kind fits the experimental result best until 30 ps. Calculation of the new annihilation constants, *k*, gives a consistent value of 4 ± 1.1 × 10^–14^ cm^3^ s^–1/2^ as shown in Figure 5c. One large outlier can be readily distinguished at the lowest fluence case, since the additional annihilation term could be overfitting. Therefore, we suggest that EEA is a time-dependent process in DPP-DTT.

**Figure 5:**
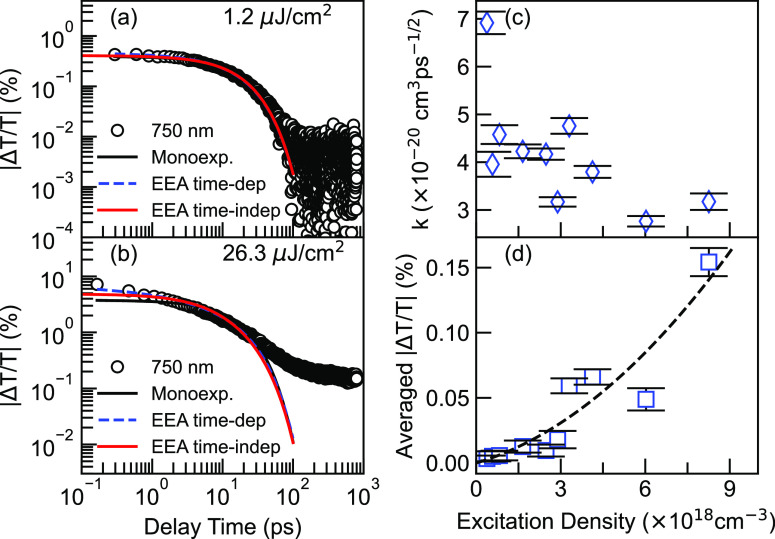
(a and b) Temporal decays under low and high fluences, respectively. The early time decays are fitted with monoexponential decay (black solid line), time-independent EEA model (red solid), and time-dependent EEA model (blue dashed line). (c) Dependence of diffusion constants on excitation densities using eq 5. (d) Time-averaged differential transmission at 800 ps with respect to excitation densities. The black dashed line is the quadratic fit with the *y*-intercept set as 0.

Another distinct feature is the drastic offset between all simulations and the experimental decay trace beyond 50 ps at the highest fluence (Figure 5b). Specifically, the long-lived tail no longer follows an exponential decay. To avoid data fluctuation at a low signal-to-noise ratio, especially in the low-fluence case, 20 points around 800 ps are averaged for each excitation density. The eventual signal at long-time delay (LTD) dependence on the excitation density is demonstrated in Figure 5d, where a quadratic dependence is observed. The corresponding density dependence is given by
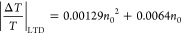
8where the *y*-intercept is set as 0 since no excited-state species should exist without a pump laser. The long-lived excited-state species likely originate from polaron pairs, and the quadratic dependence suggests EEA as the source.^6,17^ Furthermore, since eq 8 also has a linear dependence on excitation density, it also suggests that a certain amount of excitons have experienced direct dissociation. Considering the single-step exciton generation from Figure 5c, the quantum yield of the polaron pairs due to direct dissociation is estimated to be 0.7%. This value is significantly lower than in other conjugated polymer systems, where a quantum yield of 10% is estimated within the first 150 fs.^6^ One possibility could be that the quantum yield is estimated at a fairly long time delay, where a large proportion has already decayed, leading to an inaccurate estimate.

In this work, we integrate and compare the parameters acquired from both the TA and ECPL measurements based on the exciton–exciton annihilation model. As mentioned earlier, exciton–exciton annihilation can possibly be achieved by two different mechanisms, through either diffusion-limited exciton collision or direct long-range Coulombic interaction. There exists the possibility that EEA arises from long-range Coulombic interactions, assuming that the time dependence of the EEA rates originates from a spatial ensemble average of exciton interaction. However, in previous work, we showed that the exciton becomes more delocalized with increasing precursor concentration.^9^ As the exciton becomes more delocalized, the transition dipole moments weaken. The long-range Coulombic interaction would deviate from the dipole approximation to a multipole approximation (e.g., quadrupolar interactions), leading to reduced EEA. In addition, incoherent exciton hopping achieved through such Förster-type long-range interaction requires sufficient spectral overlap between the absorption and PL. For DPP-DTT, the Stokes shift increased from 130 to 180 meV with increasing precursor concentration,^9^ presumably leading to weaker EEA. Nevertheless, the opposite trend is observed, which suggests that exciton diffusion and collision might also play an important role; EEA might involve short-range interactions through either Coulombic or wave function overlap. Recently, Tempelaar et al. calculated the exciton annihilation rates theoretically, assuming that excitons interact through resonant Coulombic coupling.^46^ The annihilation rates are found to decrease with decreasing exciton densities, which is the opposite of the trend shown in Figure 4. Such evidence suggests that the annihilation between excitons through a long-range interaction might not be the active mechanism here.

It is worth mentioning that long-lived tails have been widely observed in conjugated polymers with a variety of possibilities for their origins.^8,21,38,43,47,48^ Interchain polaron pairs have been previously identified to be mediated by lattice defects with a linear dependence on pump fluence.^48^ Similar behavior might be expected for homocoupling defects due to the synthesis of DPP-based copolymers, giving rise to an unexpected lower-energy shoulder in the absorption spectra,^49^ which is nevertheless not observed in the absorption spectra of this series of samples as shown in Figure 1b. Considering the quadratic dependence on pump fluence, both possibilities can be safely excluded. Another source of the long-lived tails might be from the singlet fission of free triplet exciton and/or triplet–triplet exciton pair formation.^50,51^ In this work, we did not observe a distinct feature that can be assigned undoubtedly as triplet excitons. Besides, the triplet-exciton dependence of the fluence should also be linear since only one excited chromophore is involved in the singlet fission process. Therefore, we assign the long-lived tail as observed in this work to the polaron pairs through the EEA process, to our best knowledge.

Using the one-dimensional diffusion model, the diffusion coefficients, *D*, can be calculated based on their relation to *k*(18)
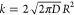
9where the annihilation radius, *R*, in the diffusion limit, is normally estimated as the lamellar layer distance, *d*_100_, as extracted from the in-plane profile of grazing incidence wide-angle X-ray scattering.^20,42^ In DPP-DTT, it is found to be around 2 nm.^52^ Therefore, the diffusion coefficient, *D*, is estimated to be 4 ± 2 nm^2^ ps^–1^ and the diffusion length is given as *L* = , which is 9 ± 2 nm. Both values are in good agreement with results found for other conjugated polymers.^10,13,44^

To compare the results with the diffusion lengths acquired from the time-independent model, we summarize the results in Table 1. The diffusion lengths acquired from the time-independent EEA model based on three-dimensional isotropic diffusion,^45^ whether from ECPL or TA, have much smaller values than those from the time-dependent model (5–10 times smaller). Such a deviation is inherited from neglecting the dimensionality of exciton diffusion. It can be simply understood as the length of the one-dimensional chain will be significantly reduced when “simulating” it into the radius of a three-dimensional sphere, considering the same volume. In addition, the diffusion lengths derived from the same time-independent EEA model differ by one time, comparing the ECPL and TA measurements. The slight difference could be due to the incorporation of the long-lived emission in ECPL measurements, as discussed earlier. Last but not least, the diffusion lengths acquired for the samples of 6 and 8 g/L are higher than those of lower concentration samples, as the diffusion is aided by the short-range interaction supported by the enhanced chain backbone order.

**Table 1:** Comparison of the Diffusion Lengths Acquired from the Time-Dependent and Time-Independent EEA Model Acquired from ECPL and TA Measurements

Conc. (g/L)	4	6	8		
Technique	ECPL	ECPL	ECPL	Time-indep. TA	Time-dep. TA
*L* (nm) at *n*_0,l_^a^	0.9 ± 0.7	1.4 ± 0.5	1.3 ± 0.6	2.6 ± 1.0	8.2 ± 0.5^c^
*L* (nm) at *n*_0,h_^b^	0.42 ± 0.08	0.49 ± 0.08	0.48 ± 0.08	0.9 ± 0.3	6.6 ± 0.4

a *n*_0,l_ and *n*_0,h_ denote initial excitation density at lowest and highest pump fluence, respectively.

b *n*_0,l_ and *n*_0,h_ denote initial excitation density at lowest and highest pump fluence, respectively.

c Value obtained for the second highest excitation density as shown in Figure 5c. The first point is ignored for its obvious deviation.

It is worth mentioning that in our current ECPL analysis, we determined the contribution from stimulated emission and/or excited-state reabsorption from the prompt PL followed by the first pump. Although it can be easily compensated for by loosening the constraint on the monoexponential decay constant, α, but its contribution should be investigated rigorously, which is outside the scope of this work. In addition, the complex eq 7 obviously prohibits us from getting a simple analytical model for ECPL measurement, as was possible with its time-independent counterpart. However, numerical methods such as a Genetic Algorithm might be one of the options for achieving a universally applicable model for extracting both monomolecular and annihilation rate constants, which can be further employed in other systems with even more complicated dynamics.^21^

In conclusion, we examine the dynamics of exciton–exciton annihilation in a specific push–pull polymer and compare the experimental and simulation results obtained from transient absorption and excitation correlation spectroscopy. Using the time-independent annihilation model, both measurements yield a decreasing annihilation rate trend with increasing fluence until they reach a plateau. Thin films deposited from higher precursor solution concentrations exhibit higher annihilation rates, likely due to stronger short-range Coulombic interactions or wave function overlap between excitons. By analyzing the time evolution of exciton density at an early stage (20 ps) in transient absorption, we find that the annihilation rate follows a *t*^–1/2^ dependence, suggesting one-dimensional exciton diffusion along the chain in DPP-DTT. The one-dimensional diffusion length is estimated to be 9 nm, which is in good agreement with a variety of other conjugated polymers. Additionally, besides the rapid decay, there is a long-lived tail that becomes more prominent as pumping fluences increase. This tail demonstrates a quadratic dependence, indicating an increasing yield of charges through exciton–exciton annihilation. Our work rigorously shows the application of the ECPL technique in conjugated polymers and a further reach into the wider semiconductor research field.

## Supporting Information Available

Experimental methods for TA and ECPL and their associated measurements and fits under varying fluences (PDF)
Transparent Peer Review report available (PDF)
