# Supplementary material for: Exciton Bimolecular Annihilation Dynamics in Push–Pull Semiconductor Polymers
Source: J Phys Chem Lett. 2024 Jan 2;15(1):272–80. doi: 10.1021/acs.jpclett.3c03094 (PMC10788955; doi:10.1021/acs.jpclett.3c03094)
Supplement: Supplementary file 1 — jz3c03094_si_001.pdf [file jz3c03094_si_001.pdf]

Supporting Information: Exciton Bimolecular Annihilation Dynamics in Push-Pull Semiconductor Polymers

Yulong Zheng[1], Rahul Venkatesh[2], Esteban Rojas-Gatjens[1], Elsa Reichmanis[3], Carlos Silva-Acuña[‡1,4]

[1] *School of Chemistry and Biochemistry, Georgia Institute of Technology, 901 Atlantic Drive, Atlanta, Georgia 30332, USA.*

[2] *School of Chemical and Biomolecular Engineering, Georgia Institute of Technology, 311 Ferst Drive NW, Atlanta GA 30332, United States.*

[3] *Department of Chemical & Biomolecular Engineering, Lehigh University, 124 E. Morton Street, Bethlehem PA 18015, United States.*

[4] *Institut Courtois & Département de physique, Université de Montréal, C.P. 6128, Succursale centre-ville, Montréal H3C 3J7, Québec, Canada.*

E-mail: carlos.silva@umontreal.ca

Contents

1 Transient Absorption Measurements **S2**

1.1 Experimental Methods S2

1.2 Experimental Results and Simulations S2

2 Excitation Correlation Photoluminescence Spectroscopy **S4**

2.1 Experimental Methods S4

2.2 Experimental Results and Simulations S4

2.3 Derivation of the time-integrated bimolecular model S7

1 Transient Absorption Measurements

1.1 Experimental Methods

Transient absorption measurements were conducted using an ultrafast laser system, specifically the Pharos Model PH1-20-02-10 by Light Conversion. Tunable wavelengths were achieved by utilizing a laser fundamental with a wavelength of 1030 nm at a repetition rate of 100 kHz. The integrated transient absorption was assessed within a commercial setup, the Light Conversion Hera system. For the pump, wavelengths spanning from 360 to 2600 nm could be adjusted by directing the 10W laser output into a commercial optical parametric amplifier known as Orpheus (manufactured by Light Conversion, Lithuania). On the other hand, the probe beam was generated by sending 2W of power through a sapphire crystal to produce a single-filament white-light continuum within the spectral range of 490-1060 nm. Following this, the probe beam, after passing through the sample, was collected using an imaging spectrograph (Shamrock 193i, Andor Technology Ltd., U.K.) combined with a multichannel detector boasting 256 pixels and covering a wavelength range from 200 to 1100 nm. The pump was selected at 730 nm while the decay traces were monitored at 750 nm. All the samples were measured in a homemade vacuum chamber at ambient temperature.

1.2 Experimental Results and Simulations

The transient absorption measurements pumped from low to high fluences for the thin film prepared from 8 g/L precursor solutions are shown in Fig. S1. The two extreme cases (1.2 and 26.3 $\mu J/cm^2$) are shown in the main article. All measurements show similar spectral features with ground bleach signals from 1.4 to 1.9 eV, while the photoinduced absorption extends below 1.4 eV.

The temporal decays at varying fluences observed at 750 nm are shown in Fig. S2. It can be clearly observed that with increasing fluences, the tails beyond 300 ps start to deviate from all three models (monoexponential, time-independent and time-dependent annihilation).

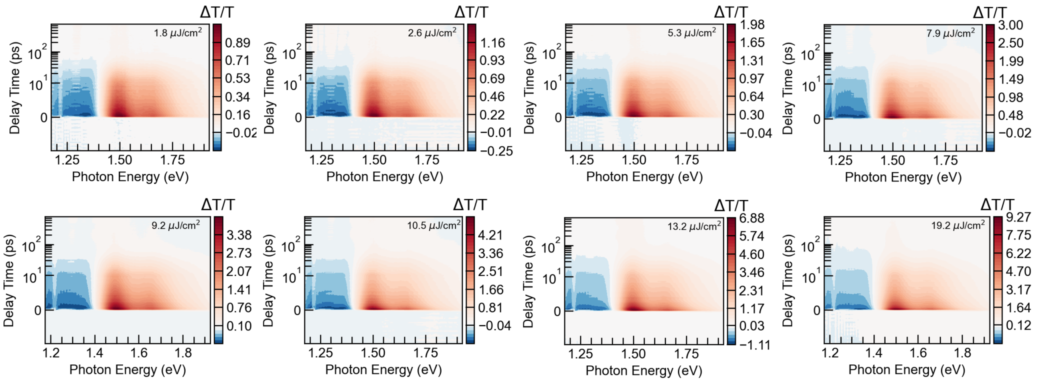

Figure S1: transient absorption maps of the 8 g/L thin film sample from the lowest to highest fluences.

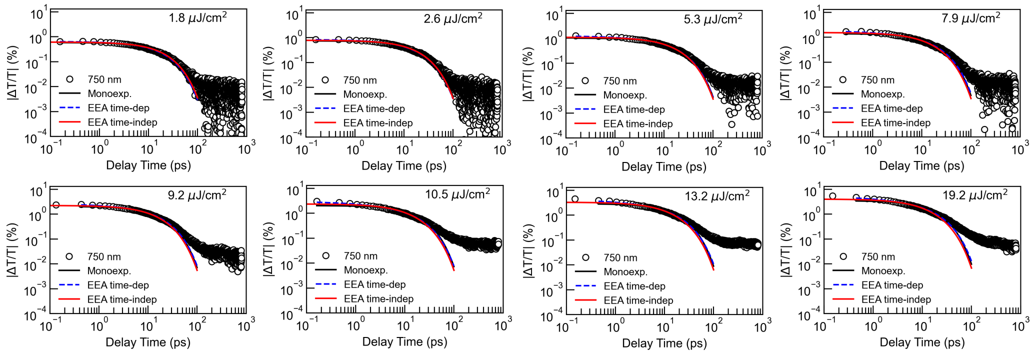

Figure S2: The temporal decay measured from low to high fluences (black open circles). The decay traces are fit with monoexponential decay (black solid line), bimolecular annihilation models with time-independent (red solid line), and -dependent bimolecular rates (blue dash line), respectively.

2 Excitation Correlation Photoluminescence Spectroscopy

2.1 Experimental Methods

A pump at a wavelength of 730 nm was generated using the previously mentioned Orpheus system. Subsequently, the beam was directed to a 50/50 beam splitter cube, where one of the beams was directed towards a motorized linear stage (LTS300, Thorlabs), enabling precise control over the time delay between the two pulses. Each pulse was modulated using a chopper at frequencies of 372 and 199 Hz, respectively, before being focused onto the sample using a 100 mm focal length lens. Both the total integrated response and the nonlinear component were simultaneously obtained by demodulating both the fundamental and the sum of the modulation frequencies. For photoluminescence detection (ECPL), the emitted PL underwent filtration with a 750 nm long-pass filter to eliminate the pump light, after which it was focused into a photoreceiver (New Focus 2031 PR) connected to a lock-in amplifier (HF2LI, Zurich Instruments). All the measurements are performed within a home-built vacuum chamber at ambient temperature.

2.2 Experimental Results and Simulations

The ECPL profiles probed for samples prepared from precursors solutions of 4, 6 and 8 g/L and their associated fits, based on Eq. 6 in the main article, are shown in Fig.S3, S4, S5, respectively. All figures show negative signals regardless the precursors' concentrations. The extracted parameters, γ and β, are shown in Fig. 4 in the main article.

The monolecular decay rates acquired from the ECPL time-independent EEA model, are shown in Figure S6, when the setting the bound loose.

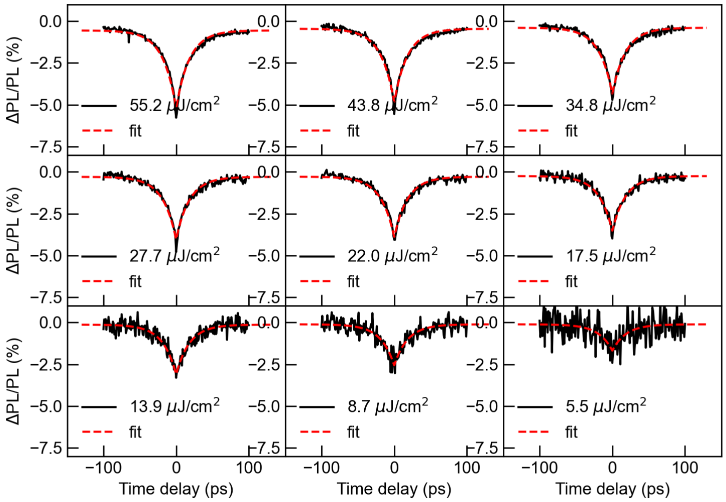

Figure S3: The ECPL profiles of 4 g/L (black solid line) and fits (red dash line) from the highest to the lowest fluences.

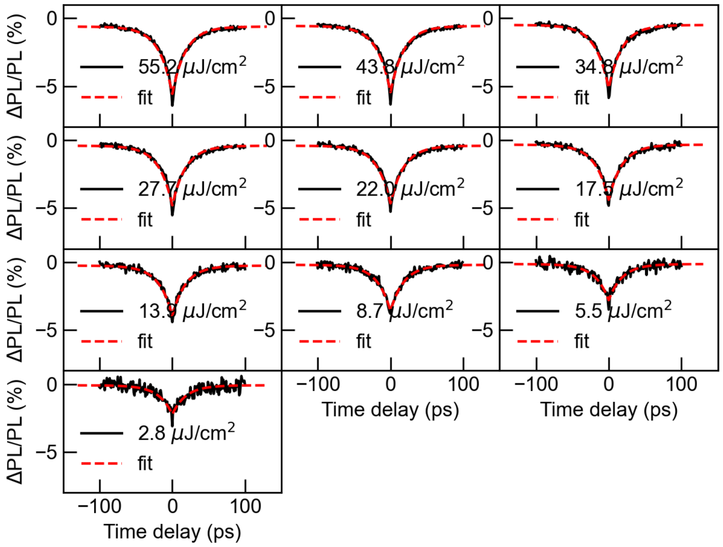

Figure S4: The ECPL profiles of 6 g/L (black solid line) and fits (red dash line) from the highest to the lowest fluences.

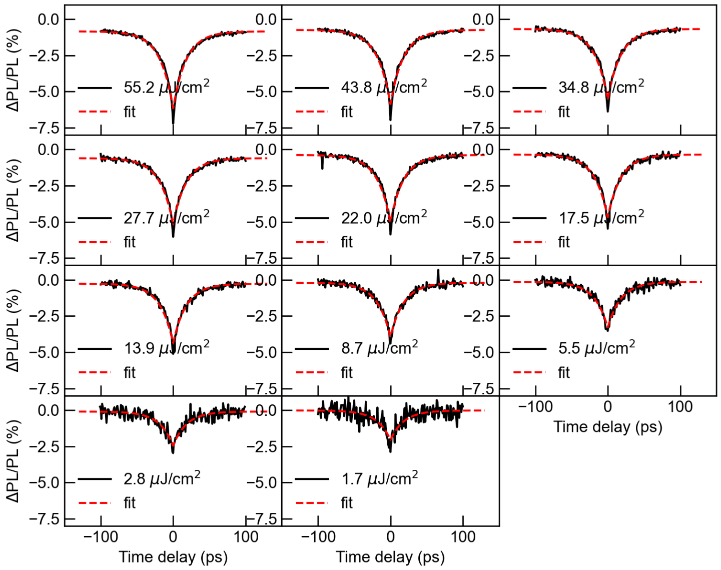

Figure S5: The ECPL profiles of 8 g/L (black solid line) and fits (red dash line) from the highest to the lowest fluences.

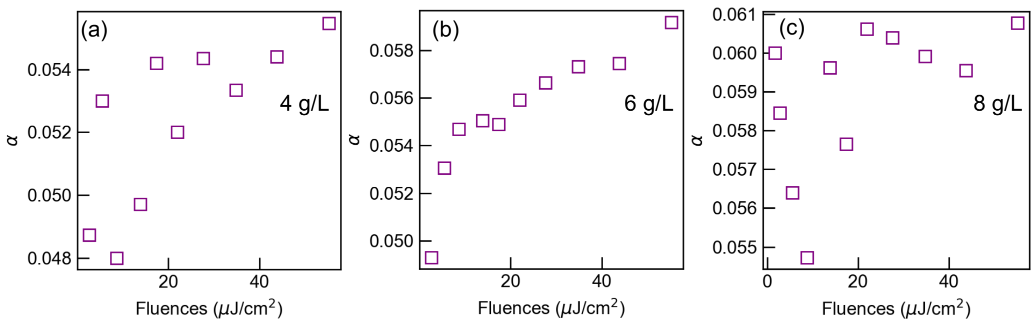

Figure S6: The monolecular decay rates α acquired from the ECPL simulations when setting the bound free for (a) 4, (b) 6, and (c) 8 g/L sample. The unit of α is (ps^{-1}).

2.3 Derivation of the time-integrated bimolecular model

We start from a simple bimolecular annihilation model, with monoexponential decay rate, α and bimolecular recombination rate, $\beta(t)$,

$$\frac{dn}{dt} = -\alpha n - \beta(t)n^2$$
$$\Rightarrow n(t) = \frac{n_0 e^{-\alpha t}}{1 + n_0 \int_0^t \beta(t)e^{-\alpha t}dt}$$

If the annihilation rate is time-independent (the diffusion is isotropic), it would give rise to,

$$n(t) = \frac{\alpha n_0}{(\alpha + n_0\beta)e^{\alpha t} - \beta n_0}$$

For individual PL demodulated at ω_1 or ω_2, it will give,

$$PL_{ind} = \int_0^\infty n(t)dt = 1/\beta\{ln[(1+\gamma)]\}$$

where $\gamma \equiv \frac{n_0\beta}{\alpha}$. Demodulate the signal at frequency ω_1+ω_2, where the ω_1, ω_2 are the chopping frequencies for each incident beam, then it gives the PL_{sum},

$$PL_{sum} = \int_0^\tau n(t_1)dt_1 + \int_0^\infty n(t_2)dt_2$$
$$= 1/\beta\{ln[(1+\gamma)^2 - \gamma^2 e^{-\alpha\tau}]\}$$

where τ is the delay time between the two beams. The final ECPL signal will be given as,

$$\Delta PL/PL(\tau) = \frac{PL_{sum} - 2 \times PL_{ind}}{PL_{sum}}$$
$$= 1 - \frac{2ln(1+\gamma)}{ln[(1+\gamma)^2 - \gamma^2 e^{-\alpha\tau}]}$$

When τ=0, it will collapse into,

$$\Delta PL/PL = 1 - \frac{2ln(1+\gamma)}{ln(2\gamma+1)}$$

The key claim that ECPL is more sensitive in probing nonlinear dynamics comes from such detection scheme. When the exciton only decays monoexponentially (i.e. γ=0), the ECPL signal will always be 0, regardless the delay time.
