# Supplementary material for: Exciton Bimolecular Annihilation Dynamics in Push–Pull Semiconductor Polymers
Source: J Phys Chem Lett. 2024 Jan 2;15(1):272–80. doi: 10.1021/acs.jpclett.3c03094 (PMC10788955; doi:10.1021/acs.jpclett.3c03094)
Supplement: Supplementary file 2 — jz3c03094_si_002.pdf [file jz3c03094_si_002.pdf]

jz-2023-030945.R1

Name: Peer Review Information for "Exciton Bimolecular Annihilation Dynamics in Push-Pull Semiconductor Polymers"

First Round of Reviewer Comments

Reviewer: 1

Comments to the Author

The authors present a collection of data on the nonlinear exciton dynamics of an alternating DPP-oligothiophene-based conjugated copolymer by (fluence-dependent) transient absorption and excitation-correlation photoluminescence spectroscopy. The data are interpreted in terms of an anisotropic exciton diffusion in the polymer films. Moreover, the occurence of a weak, long-lived (polar) component is postulated that recombines on a nanosecond timescale.

For a minor revision:

(1) Conclusions: ".... suggesting one-dimensional exciton diffusion in DPP-DTT." Please add one or two sentences discussing if the preferred direction of exciton diffusion is "on-chain" or "inter-chain".

(2) Figure 5b is not properly reproduced.

(3) Please discuss, if the long-lived component/species could be also related to the presence of structural defects (e.g.,homocoupling defects, as known from such copolymers made in STILLE-type coulings).

Reviewer: 2

Comments to the Author

The current manuscript by Silva-Acuna and co-workers is an extension of their recent work archived at arXiv.org (https://arxiv.org/abs/2303.10927). After a detailed photophysical study of a push-pull conjugated polymer in the former manuscript (accepted paper now as mentioned at arXiv.org), in this manuscript, they have studied the exciton-exciton annihilation (EEA) in this polymer in detail using fluence-dependent transient absorption (TA) and excitation-correlation photoluminescence (ECPL) spectroscopy. Most importantly, besides the rapid decay, they have observed a long-lived tail that becomes more prominent as pumping fluences increase. They proposed that the tail originates from the polaron pair which demonstrates a quadratic fluence-dependence, indicating an increasing yield of charges through EEA.

Although a vast range of experimental data is shown, the work is not novel enough to ensure publication in the Journal of Physical Chemistry Letters. Moreover, some points are not clear. Some specific queries/suggestions are listed below:

1. With increasing film concentration, there must be increasing interchain interactions and hence there is the ambient possibility of long-range interactions. The same group have shown in their earlier work that the exciton is more diffused through the polymeric backbone when the polymer films are processed at higher concentration. This increases the possibility of long-range interactions. Moreover, it is unlikely that the one-dimensional diffusion model will hold at any timescale for high-concentration films.

2. What about defects? The defect densities play a significant role in both the TA and PL behavior of a thin film. The defect density is unlikely to be the same for polymer films with different thicknesses. I am afraid that the changes in annihilation rates with different film concentrations are indicative of either different defect densities or/and long-range (interchain) interactions.

3. The long-lived tail may not be due to polaron. It can also arise because of an aggregated/agglomerated state (or collective delocalized state) (https://doi.org/10.1021/acs.jpcc.2c06093, https://doi.org/10.1021/cm102360x). What is the authors' view on that? This must be discussed carefully. Why has this signal been attributed to polarons specifically?

4. The detailed photophysics of this polymer at a longer timescale is not well understood. In the earlier work from the same group, the authors have not clarified it, stating the problem of comparable intensities of signal and noise at extremely low pump fluence. Here, they have used enough pump fluences to have a better signal-to-noise ratio. Therefore, I would request the authors to attempt deciphering the long-lived tail clearly by globally fitting (https://doi.org/10.18637/jss.v049.i03) a dataset collected at moderately low fluence (ensuring true signal). Assuming a kinetic model may help here. I would encourage them to consider the possibility of the existence of a low-energy state formed due to interchain interactions.

5. On pages 6-7, in the last paragraph starting at page 6, the authors discussed PL signals to derive Equation 3, then they use this equation for fluence-dependent temporal cuts at 750 nm from TA data. This isn't very clear. Does the 750 nm signal stem from stimulated emission or what?

6. As the work compares the EEA parameters extracted from TA and ECPL measurements, it would be nice to insert a comparison table. Otherwise, it isn't easy to follow this part. Some unnecessary equations already reported in previous literature may be shifted to the supporting information.

Reviewer: 3

Comments to the Author

In this work, the authors investigated the exciton dynamics of an electron push-pull conjugated polymer by fluence-dependent transient absorption and excitation-correlation photoluminescence spectroscopy, some theoretical simulations based on an exciton annihilation model are performed, they found a bimolecular rate constant decreasing trend for the ex-tracted annihilation rates with excitation fluence, and suggesting a nongenerous diffusion as reflective of anisotropic exciton diffusion.

This is a nice work with well theoretical and experimental consideration, which may provide some new insight into exciton diffusion in conjugated push-pull polymers.

I have following points which may be considered before accepting this manuscript for publication:

1) The “DPP-DTT” should give full name when first appear in text.

2) From my experience, high excitation intensity of pump can easily lead to photobleach or photodamage the polymer samples, I wonder how the authors handle the experiments at high laser pump intensity for such polymer samples?

3) The authors estimated the exciton diffusion length to be about~9 nm. I just think that besides the singlet exciton, the triplet state exciton should be dominated the excited state of the DPP-DTT which largely reduced exciton diffusion length, I think the authors may overestimate the exciton diffusion length, it may be largely less than 9 nm by considering the triplet exciton.

4) It is interesting with the ECPL, in the figure captions, all the ECPL spectra should give the pump wavelengths and monitored PL wavelength in maintext and SI. And also, I am interested what the ECPL can tell about the exciton diffusion or exciton annihilation? The monitored PL wavelength could overlapped with broad ESA, where the excited state can be repumped or pushed up, which can lead to speed up the ISC or lead to enhanced SE which reduce the diffusion length, how the authors consider the factors from their simulation.

5) I suggest that the authors should provide detail description of the experimental methods especially for the ECPL in SI.

Reviewer: 4

Comments to the Author

My apologies for the delay of the review. I have been busy with end of the year teaching duties and attendance of conferences.

I don't feel that there is serious deficiency in this paper but I don't think the paper should be accepted yet. Minor corrections are necessary. See below.

I feel that the authors focus only on push-pull conjugated polymers. For a paper in JPCL, the authors should elaborate on the relevance of their results to other related systems (e.g., conjugated molecular aggregates, etc).

I also feel that the paper was not clearly written. For example, the acronym DPP-DTT is mentioned on page 3 without being defined.

Figure 4b. The beta values for 4 g/L are different from other concentrations. It is unclear why beta is concentration dependent. The authors should discuss this.

Figure 4b and c. TA data typically have a number of contributions such as ground state bleach, excited state absorption, stimulated emission etc. In contrast, PL is only from the singlet excited state. Can the differences in signal origin explain the differences in the beta behavior in these 2 figures.

Figure 5a, the black solid line is invisible, blue dashed line is barely visible.

Figure 5b, parts of this figure is missing. I cannot review the quality of the data because parts of the figure are missing.

The authors should rewrite the acknowledgment section.

Author's Response to Peer Review Comments:

Dear Editor,

We thank you for sending us the referee comments. Please find attached a detailed response.

Best regards,

Carlos Silva

Reviewer(s)' Comments to Author:

Reviewer: 1

Recommendation: This paper is publishable subject to minor revisions noted. Further review is not needed.

Comments:

The authors present a collection of data on the nonlinear exciton dynamics of an alternating DPP-oligothiophene-based conjugated copolymer by (fluence-dependent) transient absorption and excitation-correlation photoluminescence spectroscopy. The data are interpreted in terms of an anisotropic exciton diffusion in the polymer films. Moreover, the occurrence of a weak, long-lived (polar) component is postulated that recombines on a nanosecond timescale. For a minor revision:

I recommend this paper for publication with minor revisions. Here are my specific comments:

Reply: We thank the reviewer for her/his critical evaluation of our manuscript, and for the positive recommendation. The specific responses are shown below in red font, and changes of the manuscript associated with Reviewer 1 comments are marked in red for the benefit of the reviewer and the editor.

Responses to reviewers' comments

1. Conclusions: "…. suggesting one-dimensional exciton diffusion in DPP-DTT." Please add one or two sentences discussing if the preferred direction of exciton diffusion is "on-chain" or "inter-chain".

Reply: We appreciate the reviewer's comment on defining the dimension of the exciton diffusion. One-dimensional exciton diffusion might be most likely achieved along the chains due to the more planarized chain backbone as shown in our previous work (*Zheng, Y., et al. 2023, Chem. Mater.*), where more efficient wavefunction overlap/transfer coupling is enabled with increasing precursor concentration. We excluded the possibility of across-chain transport because of the diminished spectral overlap between absorption and PL spectra, indicating weaker energy transfer enable by longrange Coulombic interactions. We have modified both the content and the conclusion text as suggested by the referee as follows. For clarity, we made modifications in line 19 Page 25 in Blue as it also pertains to another reviewer's comment:

"There exists the possibility of EEA suffered from long-range Coulombic interaction, assuming that the time dependence of the EEA rates originates from a spatial ensemble average of exciton interaction. However, in previous work, we showed that the exciton becomes more delocalized with increasing precursors' concentration.[9] As the exciton becomes more delocalized, the transition dipole moments would weaken. The long-range Coulombic interaction would deviate from the dipole approximation to multipole approximation (e.g. quadrupolar interactions), leading to reduced EEA. ~~For former case,~~In addition, it is commonly agreed that incoherent exciton hopping ~~is~~ achieved through such Fo¨rster-type long-range interaction ~~, which~~ requires sufficient spectral overlap between the absorption and PL. For DPP-DTT, the Stokes shift increased from 130 to 180 meV with increasing precursor concentration[9], supposedly leading to weaker EEA.~~However~~Nevertheless, the opposite trend is observed, which suggests that exciton diffusion and collision might also play an important role; EEA might involve short-range interactions through either Coulombic or wave function overlap."

The conclusion part on Page 16 line 38-48 is now modified:

"Thin films deposited from higher percursor concentrations exhibit higher annihilation rates, likely due to stronger short-range Coulombic interactions or wave function overlap between excitons. By analyzing the time evolution of exciton density at an early stage (20 ps) in transient absorption, we find that the annihilation rate follows a $t^{-1/2}$ dependence, suggesting one-dimensional exciton diffusion along the chain in DPP-DTT"

2. Figure 5b is not properly reproduced.

   Reply: We appreciate the reviewer pointing out the poor reproduction of Figure 5, which was due to a conversion error during the submission process. We reproduced the Figure 5 properly and is now shown in the main article.

3. Please discuss, if the long-lived component/species could be also related to the presence of structural defects (e.g.,homocoupling defects, as known from such copolymers made in STILLE-type couplings)

   Reply: We appreciate the reviewer's comments on the homocoupling defects that are widely presented in DPP-based conjugated polymers. One of the key features of the homocoupling defects is the presence of a low-energy shoulder besides the dominant vibronic peaks (*Hendriks, K.H., et al., 2014. JACS*). As shown in our previous work, the absorption spectra from the DPP-DTT thin films do not have an observable lowenergy side peak (*Zheng, Y., et al. 2023, Chem. Mater.*). At the end of page 15, we now add:

   "...Similar behavior might be expected for homocoupling defects due to the synthesis of DPP-based copolymers, giving rise to an unexpected lower-energy shoulder in the absorption spectra[48], which is nevertheless not observed in the absorption spectra of this series of samples as shown in Figure 1b. Considering the quadratic dependence on the pump fluence, both possibilities can be safely excluded."

Additional Questions: Urgency: Moderate

Significance: High

Novelty: High

Scholarly Presentation: High

Is the paper likely to interest a substantial number of physical chemists, not just specialists working in the authors' area of research?: Yes

Reviewer: 2

Recommendation: Reconsider as an article in The Journal of Physical Chemistry A/B/C.

Comments: The current manuscript by Silva-Acun˜a and co-workers is an extension of their recent work archived at arXiv.org (https://arxiv.org/abs/2303.10927). After a detailed photophysical study of a push-pull conjugated polymer in the former manuscript (accepted paper now as mentioned at arXiv.org), in this manuscript, they have studied the excitonexciton annihilation (EEA) in this polymer in detail using fluence-dependent transient absorption (TA) and excitation-correlation photoluminescence (ECPL) spectroscopy. Most importantly, besides the rapid decay, they have observed a long-lived tail that becomes more prominent as pumping fluences increase. They proposed that the tail originates from the polaron pair which demonstrates a quadratic fluence-dependence, indicating an increasing yield of charges through EEA. Although a vast range of experimental data is shown, the work is not novel enough to ensure publication in the Journal of Physical Chemistry Letters. Moreover, some points are not clear. Some specific queries/suggestions are listed below:

We appreciate the referee for her/his critical and valuable review of the manuscript and raising the issue of whether this work is sufficiently novel for JPCL. We consider that one of the most innovative aspects of this work is the combination of the ECPL technique, including a quantitative analysis of the ECPL profiles, and its application to push-pull conjugated polymers, in conjunction with more traditionally implemented transient absorption spectroscopy. While EEA is a topic that has been studied extensively by many authors

(including the corresponding author) over many years, the specific analysis broadening the approach with ECPL allows us to quantify these dynamics in a manner that we consider is appropriate to disseminate to the broader readership of JPCL. We therefore insist on publishing this work in JPCL. Having said that, we do appreciate the reviewer's points that consider other possible mechanisms that may be consistent with our data, and we have incorporated the discussion in the main article on the points discussed below.

1. With increasing film concentration, there must be increasing interchain interactions and hence there is the ambient possibility of long-range interactions. The same group have shown in their earlier work that the exciton is more diffused through the polymeric backbone when the polymer films are processed at higher concentration. This increases the possibility of long-range interactions. Moreover, it is unlikely that the one-dimensional diffusion model will hold at any timescale for high-concentration films. **Reply**: We appreciate the reviewer's mention of our previous work, and our efforts to connect our previous work with this report. In our earlier work, we did not attempt to explain the exciton *diffusion* in the conjugated thin films with varying concentrations, rather we focused on the exciton *delocalization* along the chain backbone, which was probed through steady-state spectroscopic techniques. Although there is a deterministic relation between the exciton delocalization and diffusion, this work does not intend to address that question, as it might require quantum dynamics simulations with the knowledge of excitonic coupling strength, dephasing rates and static disorder. Such work might be of great interest under the study of two-dimensional spectroscopy, which is out of the scope of this paper but will be addressed in a future publication using a slightly modified polymer structure. On the other hand, as the reviewer mentioned, we observed increasing interchain excitonic interaction with increasing precursor concentration. However, it is worth mentioning that such interaction originates from resonant nearest-neighbor Coulombic coupling between *transition dipole moments* on localized states. The long-

range coupling would involve Coulombic interaction between two delocalized chromophores. With more delocalized excitons, the transition dipole moments is weakened locally at individual sites, and therefore excitonic coupling is weakened locally. Over a midrange, the point dipole approximation breaks down and one must take into account the spatial extent of the exciton as we have implemented previously [J. Phys. Chem. C 2007, 111, 51, 19111–19119]. Experimentally, one of the key reason why we excluded the possibility of enhanced Fo¨rster-type long-range Coulombic interactions, also mentioned in the main article, is because we observed increasing Stark shift with increasing precursor concentration as shown in the first Figure of our previous work. With increasing concentration, the spectral overlap between absorption and PL actually decreases rather than increases. Recent theoretical work by Tempelaar, *et al.* has calculated the annihilation rates based on resonant Coulombic coupling, with respect to the excitation density. (*Tempelaar, R., et al., JPCL, 2017*) They showed that in either H or J aggregate system, the annihilation rates decrease with decreasing excitation densities, in contrast to Figure 4c as shown in the main article if we assume the EEA is due to Coulombic interaction only.

In this sense, we suggest that the difference in the annihilation rates between the low and high concentration samples might indicate short-range Coulombic interactions or wavefunction overlap.

We modified the paragraph starting from line 19 Page 25 significantly:

"There exists the possibility of EEA suffered from long-range Coulombic interaction, assuming that the time dependence of the EEA rates originates from a spatial ensemble average of exciton interaction. However, in previous work, we showed that the exciton becomes more delocalized with increasing precursors' concentration.[9] As the exciton becomes more delocalized, the transition dipole moments would weaken. The long-range Coulombic interaction would deviate from the dipole approximation to multipole

approximation (e.g. quadrupolar interactions), leading to reduced EEA. For former case,In addition, it is commonly agreed that incoherent exciton hopping is achieved through such F¨orster-type long-range interaction , which requires sufficient spectral overlap between the absorption and PL. For DPP-DTT, the Stokes shift increased from 130 to 180meV with increasing precursor concentration[9], supposedly leading to weaker EEA.HoweverNevertheless, the opposite trend is observed, which suggests that exciton diffusion and collision might also play an important role; EEA might involve short-range interactions through either Coulombic or wave function overlap. We cannot completely exclude the possibility of long-range Coulombic interaction in this work, which assumes the time dependence of the EEA rates originates from a spatial ensemble average of exciton interaction. Recently, Tempelaar *et al.* calculated the exciton annihilation rates theoretically, assuming that excitons interact through resonant Coulombic coupling.[46] The annihilation rates are found to decrease with decreasing exciton densities, which is the opposite of the trend shown in Figure 4. Such evidence suggests that the annihilation between excitons through long-range interaction might not be the mechanism considered here. ”

2. What about defects? The defect densities play a significant role in both the TA and PL behavior of a thin film. The defect density is unlikely to be the same for polymer films with different thicknesses. I am afraid that the changes in annihilation rates with different film concentrations are indicative of either different defect densities or/and long-range (interchain) interactions.

Reply: We appreciate the reviewer’s comments on defects. Specifically, the homocoupling defects are widely presented in DPP-based conjugated polymers. One of the key features of the homocoupling defects is the presence of a low-energy shoulder

besides the dominant vibronic peaks.(*Hendriks, K.H., et al., 2014. JACS*) As shown in our previous work, the absorption spectra from the DPP-DTT thin films do not have an observable low-energy side peak. (Zheng, Y., et al. 2023, Chem. Mater.)

Another evidence we want to highlight is the quadratic fluence dependence of the long-lived tail, indicating the generation of two-quasiparticle species, which structural defects might not be major contribution.

We edited on line 55 page 15:

"It is worth mentioning that long-lived tails have been widely observed in conjugated polymers with a variety of possibilities for their origins.[8,21,37,42,46,47] Interchain polaron pairs have been previously identified to be mediated by lattice defects with a linear dependence on pump fluence.[47] Similar behavior might be expected for homocoupling defects due to the synthesis of DPP-based copolymers, giving rise to an unexpected lower-energy shoulder in the absorption spectra,[48] which is nevertheless not observed in the absorption spectra of this series of samples as shown in Figure 1b. Considering the quadratic dependence on the pump fluence, both possibilities can be safely excluded."

3. The long-lived tail may not be due to polaron. It can also arise because of an aggregated/agglomerated state (or collective delocalized state) (https://doi.org/10.1021/acs .jpcc.2c06093, https://doi.org/10.1021/cm102360x). What is the authors' view on that? This must be discussed carefully. Why has this signal been attributed to po-

   larons specifically?

   Reply: We appreciate the reviewer's suggestion in bringing other possibilities for the long-lived species. However, the collective delocalized state (Frenkel exciton) is the primary excitation in conjugated polymers, and in DPP-DTT this accounts for the dominant absorption around 750nm. The most direct argument to assign the longlived

tail to polarions is due to the fact that the signal has a quadratic dependence with fluence, in contrast to the primary excitation as shown in Figure 2c in the main article, meaning that the primary excitation has to evolve into two components to produce the long-lived species. This finding is in good agreement with one of the earlier reports from Wang *et al.*(*Wang, K., et al., 2021, JPCL.*)

On the other hand, Paquin *et al.* previously showed that in high molecular-weight (M_w > 50*kg/mol*) P3HTs thin films, the intra- and intermolecular excitonic coupling result in longer exciton coherence length along the chain (4.5 thiophene units) compared to the one across the chains (2 chains).(*Paquin, F., et al., 2013, PRB*; *Paquin, F., et al., 2011, PRL*) Therefore, it is important to point out that the photophysical aggregates are really microscopic species, as opposed to the scale of polymeric aggregates.

4. The detailed photophysics of this polymer at a longer timescale is not well understood. In the earlier work from the same group, the authors have not clarified it, stating the problem of comparable intensities of signal and noise at extremely low pump fluence. Here, they have used enough pump fluences to have a better signal-to-noise ratio. Therefore, I would request the authors to attempt deciphering the long-lived tail clearly by globally fitting (https://doi.org/10.18637/jss.v049.i03) a dataset collected at moderately low fluence (ensuring true signal). Assuming a kinetic model may help here. I would encourage them to consider the possibility of the existence of a low-energy state formed due to interchain interactions.

   Reply: The possibility of low-energy states due to interchain coupling is a possibility that could be explored as suggested by the referee, but developing a kinetic model with a larger set of parameters, as also mentioned by the referee, would be required. It is not clear to us that this exercise will add much to the scope of this manuscript. We highlight that ECPL measurements are selective probes of nonlinear dynamics, and global fitting of the transient absorption and ECPL measurements already incorporates exciton dynamics over intermediate time windows.

5. On pages 6-7, in the last paragraph starting at page 6, the authors discussed PL signals to derive Equation 3, then they use this equation for fluence-dependent temporal cuts at 750 nm from TA data. This isn't very clear. Does the 750 nm signal stem from stimulated emission or what?

   Reply: We thank the reviewer for the comments. In this work, we assume the ground state bleach signal has a similar time dependence as the PL ones since only the first excited state is reached.

   For clarifications, we modified on line 37 Page 6:

   "The decay traces are further examined at 750 nm within the GSB region, where the oscillator strengths stem from the 0-0 vibronic Frenkel exciton. We assume that the primary PL and GSB share the same dynamics since only the first excited states are mostly populated. Such assumption allows the following EEA equations to be applicable to both TA and ECPL spectroscopies."

6. As the work compares the EEA parameters extracted from TA and ECPL measurements, it would be nice to insert a comparison table. Otherwise, it isn't easy to follow this part. Some unnecessary equations already reported in previous literature may be shifted to the supporting information.

   Reply: We appreciate the reviewer's suggestion on inserting the table for the comparison for the different models and techniques. Now it is added on Page 17. In addition, we added a explanatory paragraph below highlighted in blue:

   "To compare the results with the diffusion lengths acquired from the time-independent model, the results are summarized in Table1. The diffusion lengths acquired from the time-independent model, whether from ECPL or TA, have much smaller values than those from the time-dependent model (5-10 times smaller). Such deviation is inherited from neglecting the dimensionality of exciton diffusion. It can be simply understood as the length of one-dimensional chain will be significantly reduced when 'simulating' it

into the radius of a three-dimensional sphere, considering the same volume. In addition, the diffusion lengths derived from the same time-independent EEA model only differ by one time, comparing the ECPL and TA measurements. The slight difference could be due to the incorporation of the long-lived emission in ECPL measurements as discussed earlier. Last but not least, the diffusion lengths acquired for the sample of 6 and 8g/L are higher than those of lower concentration samples, as the diffusion is aided by the short-range interaction supported by the enhanced chain backbone order."

For some equations that were already reported in the previous literature, we hope to maintain them in the main article since it would be easier for readers with less related expertise to correlate the simulation results and experimental data. Also, all the parameters are easier to be defined and discussed based on the equations.

Additional Questions: Urgency: Low

Significance: Moderate

Novelty: Moderate

Scholarly Presentation: Moderate

Is the paper likely to interest a substantial number of physical chemists, not just specialists working in the authors' area of research?: Yes

Reviewer: 3

Recommendation: This paper may be publishable, but major revision is needed; I would like to be invited to review any future revision.

Comments: In this work, the authors investigated the exciton dynamics of an electron push-pull conjugated polymer by fluence-dependent transient absorption and excitationcorrelation photoluminescence spectroscopy, some theoretical simulations based on an exciton annihilation model are performed, they found a bimolecular rate constant decreasing trend for the extracted annihilation rates with excitation fluence, and suggesting a nongenerous diffusion as reflective of anisotropic exciton diffusion. This is a nice work with well theoretical and experimental consideration, which may provide some new insight into exciton diffusion in conjugated push-pull polymers.

We appreciate the referee for her/his critical review of the manuscript. We made modifications colorcoded in orange for the reviewer and editor's convenience on tracking our

edits.

1. The "DPP-DTT" should give full name when first appear in text.

   Reply: We appreciate the reviewer's comment and now we add the full name of DPP-DTT in the main article.

   On Page 3 line 39:

   "the push-pull conjugated polymer, DPP-DTT, (poly[2,5-(2-octyldodecyl)-3,6-diketopyrrolopyrrole-*alt*-5,5-(2,5-di(thien-2-yl)thieno-[3,2-b]-thiophene)])"

2. From my experience, high excitation intensity of pump can easily lead to photobleach or photodamage the polymer samples, I wonder how the authors handle the experiments at high laser pump intensity for such polymer samples?

Reply: Thanks for the reviewer's comment. All measurements were performed in a vacuum chamber to avoid direct polymer heating with oxygen. Although there might be local heating issue under significantly high laser pump intensities, the results are repeatible even the same samples were measured for multiple times.

Now we added the measurement condition in the SI as following: "A pump at a wavelength of 730 nm was generated using the previously mentioned Orpheus system. Subsequently, the beam was directed to a 50/50 beam splitter cube, where one of the beams was directed towards a motorized linear stage (LTS300, Thorlabs), enabling precise control over the time delay between the two pulses. Each pulse was modulated using a chopper at frequencies of 372 and 199 Hz, respectively, before being focused onto the sample using a 100 mm focal length lens. Both the total integrated response and the nonlinear component were simultaneously obtained by demodulating both the fundamental and the sum of the modulation frequencies. For photoluminescence detection (ECPL), the emitted PL underwent filtration with a 750nm long-pass filter to eliminate the pump light, after which it was focused into a photoreceiver (New Focus 2031 PR) connected to a lock-in amplifier (HF2LI, Zurich Instruments). All the measurements are performed within a home-built vacuum chamber at ambient temperature."

3. The authors estimated the exciton diffusion length to be about 9nm. I just think that besides the singlet exciton, the triplet state exciton should be dominated the excited state of the DPP-DTT which largely reduced exciton diffusion length, I think the authors may overestimate the exciton diffusion length, it may be largely less than 9 nm by considering the triplet exciton.

   Reply: We appreciate the reviewer's comment on triplet excitons. Previous literature claimed that there were two types of triplet species, free triplet excitons and

triplettriplet exciton pair. For the first type, their associated energy should be only half of the primary excitation energy (1.65eV), which is outside our detection range for either TA and ECPL. For the second type, Huynh, *et al* previously assigned one of the photoinduced feature (around 0.8eV) to triplet exciton pairs in one type of electron push-pull conjugated polymers (PDTP-DFBT). (*Huynh, U.N.V., et al., PRL, 2017*) As we are monitoring the dynamics at ground-state bleaching in TA, the contribution should not be significant. However, for the ECPL profiles, besides the possibility of the triplet excitons, there could be contributions from delayed fluorescence from polaron pairs as mentioned in the main article. For the current work, we did not incorporate the contributions for such long-lived contributions, but they would be of great interest in the future work.

On the Page 16 in the main article, we added the following:

"Another source of the long-lived tails might be from the singlet fission of free triplet exciton and/or triplet-triplet exciton pair formation.[49,50] In this work, we did not observe distinct feature that can be assigned undoubtedly as triplet excitons. Besides, the triplet-exciton dependence of the fluence should also be linear since only one excited chromophore is involved in the singlet fission process. Therefore, we assign the long-lived tail as observed in this work to the polaron pairs through EEA process, to our best knowledge."

4. It is interesting with the ECPL, in the figure captions, all the ECPL spectra should give the pump wavelengths and monitored PL wavelength in main text and SI. And also, I am interested what the ECPL can tell about the exciton diffusion or exciton annihilation? The monitored PL wavelength could be overlapped with broad ESA, where the excited state can be repumped or pushed up, which can lead to speed up the ISC or lead to enhanced SE which reduce the diffusion length, how the authors consider the factors from their simulation.

Reply:

1. On the caption of Figure3, we made the following modifications to incorporate the pump wavelengths and monitored PL wavelengths:

"ECPL nonlinear response profiles excited at 730 nm pump for samples prepared from 4 (a), 6 (b) and 8g/L (c) precursor solutions.The PL signals were filtered to collect the wavelength range of 750-1100nm. The measurements are performed under a variation of excitation densities coded by the colorbar scale."

2. We appreciate the reviewer's comment on the ECPL technique. As shown in the manuscript, the ECPL signal originated from the difference between the prompt linear PL and the cross PL, where the total PL is quenched due to the exciton-exciton annihilation. Thus, the ECPL profile directly shows the nonlinear dynamics. As it still probes the EEA, parameters could be extracted from the experimental results to describe the exciton diffusion in a way similar to that shown in TA. The real difficulty is explaining the experimental results quantitatively. This work shows that in one of the simplest senario, time-independent EEA, there exists an analytical solution for describing the dynamics, although with more complex kinetics equation, numerical simulations might be needed.

3. We really appreciate the reviewer's comment on the excited state reabsorption. This is an excellent question and concern. In ECPL, we assume the dominant contributions originate from incoherent population mixing, meaning that the two sequential pumps excite two sets of ground-state molecules. Indeed, as suggested by the reviewer, there could be excited state reabsorption and/or stimulated emission. However, in the technique of the ECPL, the analysis of the diffusion length is less dependent on the monoexponential decay rate α at least in the isotropic diffusion model. The reason lies on Eq.6, showing that the determination of the annihilation rates is based on the determination of γ, as defined $\gamma = \frac{n_0\beta}{\alpha}$. Therefore, $\beta = \alpha\gamma/n_0$. Substituting

the equation into 3D-diffusion equation $D = \frac{\beta_{3D}}{8\pi R}$ and the diffusion length expression gives,

$$L_D = \sqrt{D/\alpha} = \sqrt{\frac{\gamma}{8\pi R n_0}} \tag{1}$$

In this sense, we expect the change of α due to SE or excited state reabsorption (ESRA) might not be the key contribution to the diffusion length.

Nevertheless, the contribution from SE or ESRA can be easily compensated by loosing the constraint on the monoexponential decay α. Currently, the α is set as the value from the lowest TA measurement, assuming no significant contribution from SE or ESRA or other linear attributes. In addition, we have also looked at the case where we loosed the constraint on α. With slight increase on α (from 0.058 to 0.061ps^{-1} for 8g/L), γ maintained almost constant. We now added a new figure in the SI with the senario where the constraints on the monoexponential decay is loosed. Again we thank the reviewer for bringing this concern, and would like to add more explanations in the main article.

On Page 12 line 41:

"On the other hand, the effective monomolecular lifetime would shorten due to stimulated emission or excited state absorption with fluences, their variations are much smaller in contrast to the multiple times change of γ. (See Figure S6 in SI) ~~should be regarded as a time-invariant value in that it is intrinsic to the materials~~."

On Page 16 before line 22, we add the following modifications:

"It is also worth mentioning that in our current ECPL analysis, we ignored the contribution from stimulated emission and/or excited state reabsorption from the prompt PL followed by the first pump. Although it can be easily compensated for by loosing the constraint on the monoexponential decay constant, α but its contribution should be investigated rigorously which is outside the scope of this work."

5. I suggest that the authors should provide detail description of the experimental methods especially for the ECPL in SI.

Reply: We thank the reviewer for the comment. Now the detailed ECPL experimental method is added in the SI. The detailed ECPL experimental is shown in the repsonse to the second comment.

On Page 9 line 13 we now removed the reference to another work:

"A detailed description of the ECPL setup can be found in the SI ~~of Ref.34~~."

Additional Questions: Urgency: High

Significance: High

Novelty: High

Scholarly Presentation: High

Is the paper likely to interest a substantial number of physical chemists, not just specialists working in the authors' area of research?: Yes

Reviewer: 4

Recommendation: I don't feel that there is serious deficiency in this paper but I don't think the paper should be accepted yet. Minor corrections are necessary.

Comments:

Reply: We appreciate the reviewer for her/his critical evaluation of our manuscript, and for the positive recommendation. The specific comments are shown below.

Responses to reviewers' comments

1. I feel that the authors focus only on push-pull conjugated polymers. For a paper in JPCL, the authors should elaborate on the relevance of their results to other related systems (e.g., conjugated molecular aggregates, etc).

   Reply: We appreciate the reviewer's comment and agree with the reviewer's suggestion. Indeed, we studied the EEA in push-pull conjugated polymer due to their unique electronic structure originating from the mixing of FE and charge-transfer excitons. Now, we include a few sentences of comparison between the conjugated homopolymers and push-pull conjugated polymers.

   On page 5 line 14, we added:

   "Compared to the conjugated homopolymers, conjugated push-pull polymers inherit strong charge-transfer character due to the differences in the electronegativities of the electron-deficient and -sufficient domains, which could have another contribution for the driving force of EEA.[35]"

2. Figure 5b is not properly reproduced.

   Reply: We appreciate the reviewer's catch on Figure 5b. Now the whole figure 5 is reproduced.

3. Figure 4b. The beta values for 4 g/L are different from other concentrations. It is unclear why beta is concentration dependent. The authors should discuss this.

   Reply: We appreciate the reviewer's comment on the difference between the annihilation rates. In one of our recent work, we studied the photophysics of this series of DPPDTT conjugated polymer thin films with varying precursors' concentration.(*Zheng, Y, et al., 2023, Chem. Mater.*) With increasing concentration, we observed increasing excitonic interchain interactions as well as more delocalized exciton wave along the polymer chain. The view we held was that due to the increasing concentration, the backbone torsion of the polymer chain was reduced, leading to more planarized chain backbone. There might be also increasing interchain excitonic interactions due to Coulombic interactions. Following this, the exciton should be more localized in low-concentration sample than higher-concentration ones. As mentioned in the main article, two mechanisms were previously proposed to explain the annihilation process: one is that the annihilation is achieved through Fo¨rster-type long-range Coulombic interaction. Due to the random spatial distribution of excitons, the ensemble-averaged annihilation rates will decrease with time. Another model considers the anisotropy of exciton diffusion and excitons can only interact when they are in proximity, either through short-range Coulombic interaction or wavefunction overlap. As mentioned above, there might be enhanced long-range interactions inherited from the resonant

   Coulombic interaction. However, from the previous work (Figure 1) (*Zheng, Y, et al., 2023, Chem. Mater.*), we observed less spectral overlap between the absorption and PL with increasing concentration, indicating the Fo¨rster-type long-range might not be the dominant contributor. We therefore point to the possiblity of short-range Coulombic or wavefunction overlap which drives the exciton diffusion.

On Page 15 line 25 we added the following discussion in blue:

“There exists the possibility of EEA suffered from long-range Coulombic interaction, assuming that the time dependence of the EEA rates originates from a spatial ensemble average of exciton interaction. However, in the previous work, we showed that the exciton become more delocalized with increasing precursors' concentration.[9] As the exciton become more delocalized, the transition dipole moments would weaken. The long-range Coulombic interaction would deviated from dipole approximation to multipole approximation (e.g. quadrupolar interactions), leading to reduced EEA.”

4. Figure 4b and c. TA data typically have a number of contributions such as ground state bleach, excited state absorption, stimulated emission etc. In contrast, PL is only from the singlet excited state. Can the differences in signal origin explain the differences in the beta behavior in these 2 figures.

   Reply: We thank the reviewer for the comment. In Figure 4b and c, we specifically monitored the time decay at 750nm, which is at the center wavelength for ground state bleaching only. We assume the GSB dynamics has a similar time dependence as the prompt PL because the only the first excited state is reached.

   We highlighted this assumption by added the following sentence on Page 6 line 40:

   “We assume that the primary PL and GSB share the same dynamics since only the first excited states are mostly populated. Such assumption allows the following EEA equations to be applicable to both TA and ECPL spectroscopies.”

5. Figure 5a, the black solid line is invisible, blue dashed line is barely visible. **Reply**: We thank the reviewer's comment on the overlap between the three kinetic model. Indeed, the key point we would like to highlight here is that the dominant GSB dynamics can be simply explained by a single monoexponential decay at low pump fluence, although both the time-dependent and -independent model could also simulate the kinetics due to the additional parameter it introduces.

   To highlight this, we edited the sentences on Page 13 line 23:

”Under the lowest pumping fluence, all three models fit the dynamics closely indicating that the dynamics at low pump fluence is dominated by monoexponential decay with minor impact from EEA. However, under high pump fluence, a small deviation becomes clear in the early delay times (first 2ps) when comparing the time-dependent annihilation model with the other two; the first kind fits the experimental result best till 30ps. ”

6. Figure 5b, parts of this figure is missing. I cannot review the quality of the data because parts of the figure are missing.

   Reply: We sincerely apologize for the missing part. This whole figure is now reproduced.

7. The authors should rewrite the acknowledgment section.

   Reply: We appreciate the reviwer’s comment on the acknowledgement session, now it is rewritten.

Per the editor's comments:

1. Please submit your publication files without any markups. Any copies that contain highlights, colored text, or tracked changes should be submitted as "Supporting Information for Review Only."

2. Abstract: Shorten the abstract to 150 words or fewer.

   We appreciate the editor's remind on the abstract limit. Now the edits are made in brown.

3. References: In both the main file and the supporting information, fix the style of all references to use JPCL formatting (check all references carefully). ***JPC Letters reference formatting requires that journal references should contain: () around numbers, author names, article title (titles entirely in title case or entirely in lower case), abbreviated journal title (italicized), year (bolded), volume (italicized), and pages (first-last). Book references should contain author names, book title (in the same pattern), publisher, city, and year. Websites must include date of access.

   We appreciate the editor's comment and we made the adjustments on the reference formats accordingly.

In addition to the comments mentioned above, we also made slight modifications on certain wordings, grammers and missing citations highlighted in brown.
